# Prepackaged Low‐Residue Diet “Clear‐Through” Reduces the Required Volume of Polyethylene Glycol Solution for Colonoscopy Preparation: An Exploratory Randomized Controlled Study

**DOI:** 10.1002/deo2.70332

**Published:** 2026-04-28

**Authors:** Toshifumi Iida, Yoshiaki Kimoto, Etsuko Yamabe, Miuzen Kanamori, Susumu Banjoya, Tomoya Kimura, Koichi Furuta, Shinya Nagae, Hiroshi Yamazaki, Nao Takeuchi, Shunya Takayanagi, Yuki Kano, Kohei Ono, Ryoju Negishi, Yohei Minato, Hideyuki Chiba, Ken Ohata

**Affiliations:** ^1^ Department of Gastrointestinal Endoscopy NTT Medical Center Tokyo Tokyo Japan; ^2^ Division of Gastroenterology, Itabashi Chuo Medical Center Tokyo Japan; ^3^ Department of Medicine Haukeland University Hospital Bergen Norway; ^4^ Department of Gastroenterology Omori Red Cross Hospital Tokyo Japan

**Keywords:** bowel preparation, colonoscopy, exploratory study, low‐residue diet, PEG‐Asc

## Abstract

**Objectives:**

Large volumes of polyethylene glycol (PEG) solution reduce patient acceptance of colonoscopy. A prepackaged low‐residue diet (PLRD; Clear‐Through) may improve bowel cleansing and allow further PEG reduction. This exploratory randomized study evaluated whether PLRD permits decreased PEG with ascorbic acid (PEG‐Asc) volume while maintaining cleansing quality.

**Methods:**

In this single‐center, endoscopist‐blinded, three‐arm randomized trial, 180 patients were allocated to Group A (self‐adjusted diet + 1.5 L PEG‐Asc), Group B (PLRD + 1.5 L PEG‐Asc), or Group C (PLRD + 1.0 L PEG‐Asc). The primary endpoint was cleansing quality using the Ottawa Bowel Preparation Score (OBPS). Secondary endpoints included bubble score, bowel movement frequency, adherence, tolerability, patient satisfaction compared with the previous colonoscopy, and adverse events. Logistic regression identified predictors of inadequate cleansing.

**Results:**

Baseline characteristics were comparable across groups. OBPS didn't differ significantly among the groups. Group C had significantly fewer bowel movements (*p* < 0.001) and a lower bubble score (*p* < 0.001). Adherence and tolerability were high, with no major adverse events. Reduced‐volume preparation didn't negatively affect patient satisfaction; Group C had the highest proportion rating the preparation better than their previous experience (42.9%; *p* = 0.019). PLRD intake independently reduced the risk of inadequate right‐colon cleansing (odds ratio 0.119; 95% confidence interval 0.043–0.327; *p* < 0.001).

**Conclusions:**

PLRD allowed reduction of PEG‐Asc to 1.0 L without impairing cleansing quality, while maintaining favorable patient satisfaction. PLRD may serve as a practical strategy to lessen patient burden during colonoscopy preparation.

**Trial Registration:**

UMIN 000056459.

## 1 Introduction

The increasing demand for colonoscopy, driven by the rising prevalence of colorectal cancer and inflammatory bowel disease [1, 2, 3, 4, 5, 6], highlights the importance of effective bowel preparation. However, standard polyethylene glycol (PEG)‐based regimens require large volumes and are often poorly tolerated [7, 8, 9, 10, 11], leading to reduced patient compliance. Bowel preparation can be burdensome for patients and may decrease the uptake rate of colorectal cancer screening [12, 13, 14]. Reducing this burden could reduce the overall discomfort of colonoscopy. In this exploratory randomized study, we evaluated whether a prepackaged low‐residue diet (PLRD; Clear‐Through, Kewpie Corp., Tokyo, Japan) combined with a reduced volume of PEG with ascorbic acid (PEG‐Asc; Moviprep, EA Pharma Co., Ltd., Tokyo, Japan) could maintain adequate bowel cleansing while improving patient satisfaction and tolerability.

## 2 Patients/Materials and Methods

### 2.1 Study Design

This study was a single‐center, endoscopist‐blinded, three‐arm exploratory randomized trial. Patients were randomized in a 1:1:1 ratio into three groups using a computer‐generated allocation sequence within the institutional clinical research system. The allocation sequence was generated prior to patient enrollment and was not accessible to the endoscopists performing the procedures. The endoscopists who evaluated bowel preparation quality were therefore blinded to group allocation. Patients undergoing colonoscopy between April and December 2024 at NTT Medical Center Tokyo in Japan were included. Written informed consent was obtained from each patient. The study was conducted with the approval of the Institutional Review Board of NTT Medical Center, Tokyo (IRB No. 000200016279‐01) and was registered with the University Hospital Medical Information Network (UMIN) Clinical Trials (www.umin.ac.jp/ctr/; identification No. UMIN 000056459). All authors had access to the study data and reviewed and approved the final manuscript.

### 2.2 Patients

We enrolled 180 patients aged 20–79 years who underwent total colonoscopy at our hospital between April and December 2024 and randomly assigned them in a 1:1:1 ratio to three preparation groups (*n* = 60 each) using a computer‐generated allocation sequence within our institutional clinical research system. Because the intervention involved dietary intake, blinding of patients was not feasible; however, endoscopists who evaluated bowel preparation quality using the Ottawa Bowel Preparation Score (OBPS) were blinded to group allocation. Group A received a self‐adjusted diet and sodium picosulfate, followed by 1.5 L of PEG‐Asc. Groups B and C received a PLRD with sodium picosulfate, followed by 1.5 L and 1.0 L of PEG‐Asc, respectively. PEG‐Asc was administered as a split‐dose regimen approximately 3 h before colonoscopy, with all procedures performed in the afternoon. Intake followed a 2:1 PEG‐Asc‐to‐water ratio, with an additional 0.5 L administered if needed. All preparation was conducted after hospital arrival under medical supervision. This PLRD was designed to be compatible with the typical Japanese diet and consisted of three meals on the day before colonoscopy: chicken soboro and egg rice porridge for breakfast; potato soboro ankake with plain rice porridge for lunch; and beef stew with crackers for dinner. One PLRD set contained 690 kcal, 24 g of protein, 22.4 g of fat, 100.7 g of carbohydrates, 4.6 g of dietary fiber, 4.0 g of salt, 273 mg of phosphorus, and 748 mg of potassium. The cost of one PLRD set was approximately 10 US dollars.

The exclusion criteria were: 1) patients who had received preoperative chemotherapy, 2) patients with insufficient clinical information, 3) age >79 years or <20 years, 4) history of colectomy, 5) history of bowel obstruction, 6) severe constipation (passage of less than three stools per week), 7) current pregnancy or breastfeeding, 8) body mass index (BMI) >30, and 9) allergies to cleansing agents or ingredients of PLRD.

### 2.3 End Points and Outcome Evaluation

The primary endpoint of this study was bowel cleansing quality, which was evaluated using the OBPS [15]. Secondary endpoints included the amount of residual bubbles, scored on a 0–6 scale; patient satisfaction, categorized as “better,” “equal,” or “worse” compared with previous colonoscopy experience; indicators of tolerability such as abdominal discomfort and completion rate; and the occurrence of adverse events. The amount of intraluminal bubbles was evaluated across three predefined colonic segments: the cecum and ascending colon; the transverse and descending colon; and the sigmoid colon and rectum. Each segment was graded on a three‐point scale according to the extent to which bubbles interfered with mucosal visualization. A score of 0 indicated minimal or no bubbles and no need for removal, whereas a score of 1 indicated the presence of bubbles that required only minimal effort to clear and did not impede examination. A score of 2 was assigned when abundant bubbles were present, and substantial removal effort was needed, thereby interfering with the progression of the procedure. The total bubble score ranged from 0 to 6 and was calculated as the sum of the three segmental scores. The performing endoscopists were blinded to group allocation, and OBPS scoring was conducted without knowledge of the dietary regimen or assigned PEG‐Asc volume. All OBPS assessments were therefore conducted under blinded conditions to minimize potential evaluation bias. The quality of each subject's bowel preparation was scaled or scored by endoscopists experienced in more than 2000 endoscopies after the examination. Inadequate bowel preparation was defined as an OBPS ≥5 for the entire colon, applying a stricter definition of suboptimal cleansing. Inadequate right‐colon cleansing was defined as an OBPS ≥3 for the cecum and ascending colon.

Patient adherence, tolerability, and overall impressions of bowel preparation were assessed using a structured questionnaire administered prior to colonoscopy. The questionnaire collected information on prior colonoscopy experience, usual bowel habits, and hunger after the low‐residue diet, as well as evaluations of the taste and volume of the diet and PEG‐Asc solution. Participants also reported the actual volume of PEG‐Asc ingested and any associated discomfort or difficulties. Adverse events were classified as major if medical attention was required and minor if self‐limited. In patients with prior colonoscopy, satisfaction and convenience were assessed relative to previous preparation.

### 2.4 Sample Size Justification and Statistical Analysis

Because this was an exploratory randomized study, no formal sample size calculation was performed. Instead, the target enrollment of 60 patients per group was determined pragmatically, based on feasibility considerations and consistency with the sample sizes used in prior randomized trials evaluating PLRDs and PEG‐Asc regimens. These earlier studies generally enrolled similar numbers of participants per arm while demonstrating acceptable bowel cleansing quality [16, 17, 18]. Continuous variables were expressed as means ± standard deviation or medians, and were analyzed using Student's t‐test or the Mann‐Whitney U test for two‐group comparisons, and analysis of variance or the Kruskal‐Wallis test for three‐group comparisons, as appropriate. Categorical variables were described as frequencies (percentages) and compared using the chi‐square test or Fisher's exact test. Logistic regression analysis was performed to identify factors associated with inadequate bowel cleansing. All statistical analyses were conducted using SPSS version 25.0 (IBM Corp., Armonk, NY, USA). A *p*‐value <0.05 was considered statistically significant. Multivariable logistic regression analysis was performed to identify factors associated with inadequate bowel preparation. The following variables were included in the model: age, gender, BMI, previous colonoscopy, bowel movement less than once daily, presence of colonic diverticula, reduction of PEG‐Asc volume, and intake of a PLRD.

## 3 Results

### 3.1 Patient Enrollment and Baseline Characteristics

**FIGURE 1:**
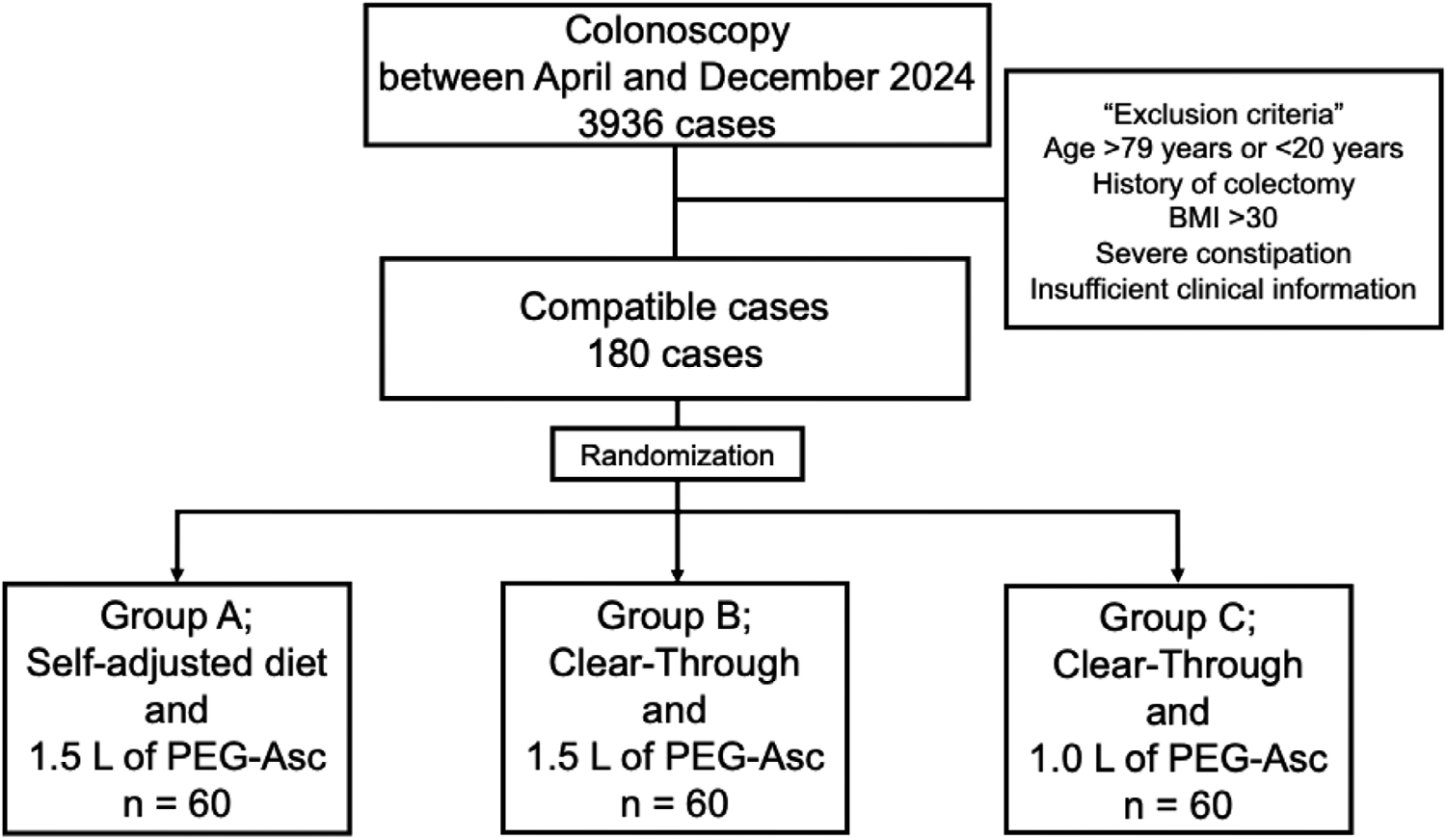
Flowchart of patient selection and randomization. Among 3936 colonoscopy cases screened, 180 patients met eligibility criteria and were randomized equally into three groups: Group A (self‐adjusted diet + 1.5 L PEG‐Asc), Group B (Clear‐Through + 1.5 L PEG‐Asc), and Group C (Clear‐Through + 1.0 L PEG‐Asc). BMI, Body Mass Index.

A total of 3936 patients aged 20–79 years underwent colonoscopy at our institution between April and December 2024. After applying the prespecified exclusion criteria—age outside the eligible range, history of colectomy, severe constipation, BMI >30, or insufficient clinical information – 180 patients who provided informed consent were enrolled in the study. These participants were randomly assigned in equal numbers to three groups (*n* = 60 each) and were included in the comparative analysis (Figure 1).

Baseline characteristics were comparable among the three groups (Table 1). The prevalence of diverticula showed no notable variation. Indications for colonoscopy were similar across groups, with screening being the most common. Overall, the randomized groups were considered well‐matched at baseline.

**TABLE 1:** Baseline characteristics of the study population.

	Total	A	B	C	*p*‐Value
Age, years, mean (SD)	57 ± 11.1	56 ± 11.3	58 ± 10.7	56 ± 11.3	0.465
Gender, male, %	73.3	73.3	66.7	80.0	0.256
BMI, kg/m^2^, mean (SD)	24.2 ± 4.24	24.0 ± 4.87	23.4 ± 3.50	23.2 ± 4.20	0.389
Previous colonoscopy, %	69.4	61.7	76.7	70.0	0.202
Bowel movement less than once daily, %	81.7	83.3	78.3	83.3	0.716
Diverticulum, %	21.7	16.7	23.3	25.0	0.503
Indication for colonoscopy, %					0.932
Screening	62.2	66.7	60.0	60.0	
Surveillance	15.0	13.3	16.7	15.0	
Clinical symptoms	22.8	20.0	23.3	25.0	

Baseline demographic and clinical characteristics were comparable among the three groups. No significant differences were observed in age, sex distribution, BMI, previous colonoscopy history, bowel movement frequency, presence of diverticula, or indication for colonoscopy.

### 3.2 Adherence, Tolerability, and Patient Satisfaction

Adherence to both the dietary instructions and PEG‐Asc ingestion was excellent across all groups. Nearly all participants completed the assigned regimen, and deviations from the instructed PEG‐Asc volume were minimal. Abdominal discomfort related to bowel preparation was uncommon, occurring in only 6.1% of participants overall, with no significant differences among the groups. Importantly, no major adverse events occurred, and no patients required unplanned medical visits during the preparation period. Minor issues were rare and self‐limiting. Additional PEG‐Asc (0.5 L) was administered when necessary, and the proportion of patients requiring additional dosing is shown in Table 2. The mean total volume of PEG‐Asc actually ingested was approximately 1.53 L in Groups A and B and 1.02 L in Group C.

**TABLE 2:** Adherence and tolerability outcomes.

	Total	A	B	C	*p*‐Value
Abdominal discomfort, %	6.1	8.3	5.0	5.0	0.679
Major events, %	0.0	0.0	0.0	0.0	1.000
Minor events other than abdominal discomfort, %	0.0	0.0	0.0	0.0	1.000
Additional PEG‐Asc, %	6.7	5.0	5.0	3.3	0.877
Completed low‐residue diet, %	99.2	—	98.3	100	—
Participants who felt the low‐residue diet tasty, %	85.0	—	83.3	86.7	—
Participants who felt the low‐residue diet sufficient, %	88.3	—	85.0	91.7	—

Adherence to the assigned bowel preparation regimen and tolerability were comparable among the three groups. Rates of abdominal discomfort were low, and no major or minor adverse events were observed. The need for additional PEG‐Asc was infrequent and did not differ significantly across groups. Completion of the prepackaged low‐residue diet was high in both Groups B and C, and more than 80% of participants reported that the diet was sufficiently palatable and adequate in quantity.

Adherence to the PLRD was similarly high. Most participants completed the provided meals, and more than 80% reported that the taste and quantity of the diet were acceptable or sufficient (Table 2).

**FIGURE 2:**
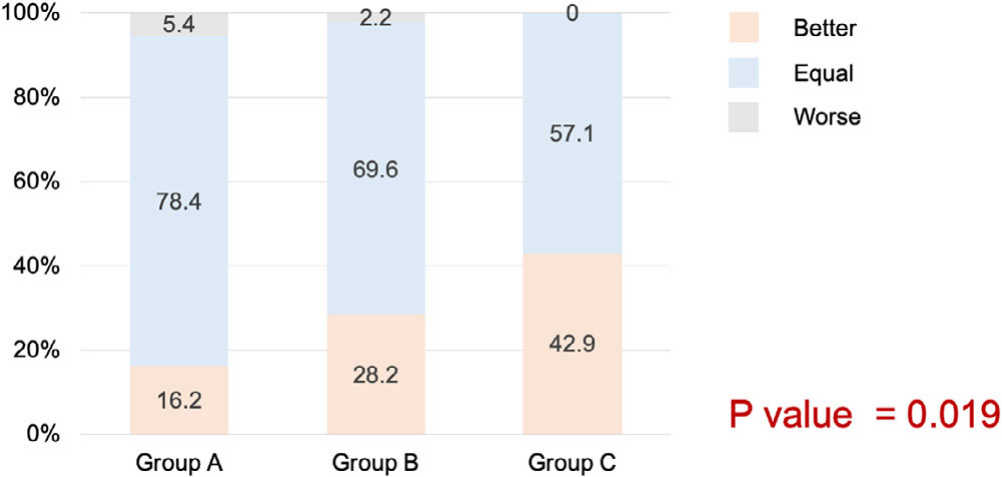
Patient satisfaction with bowel preparation compared with previous colonoscopy experience. Patient‐reported satisfaction differed significantly among the three groups (*p* = 0.019). A higher proportion of participants in Group C reported that the current preparation was “better” compared with their previous colonoscopy, whereas the majority in Groups A and B rated their experience as “equal.” Only a small proportion in any group reported a “worse” experience.

Patient satisfaction showed a clear gradient across the three groups. Compared with their previous colonoscopy experience, a higher proportion of participants in Groups B and C—both of which received the prepackaged diet—reported improved satisfaction with the current preparation. The percentage of participants who rated the preparation as “better” was 16.2% in Group A, 28.2% in Group B, and 42.9% in Group C, resulting in a significant intergroup difference (*p* = 0.019). Dissatisfaction (“worse”) was nearly absent in all groups, indicating that all regimens were generally acceptable, although the combination of a prepackaged diet and reduced‐volume PEG‐Asc provided the most favorable overall experience (Figure 2).

### 3.3 Cleansing Efficacy

Cleansing efficacy outcomes are summarized in Table 3. The overall quality of bowel cleansing, as assessed by the OBPS, did not differ significantly among the three groups. Mean OBPS values were comparable among the groups.

**TABLE 3:** Cleansing efficacy outcomes among the three preparation groups.

	Total	A	B	C	*p*‐Value
Frequency of bowel movements, mean (SD)	10.3 ± 2.43	10.9 ± 2.20	11.0 ± 2.38	8.9 ± 2.14	<0.001
OBPS, mean (SD)	3.3 ± 1.18	3.6 ± 1.11	3.1 ± 1.16	3.3 ± 1.24	0.086
Amount of bubble (0–6), mean (SD)	1.8 ± 1.17	2.3 ± 1.07	1.4 ± 1.01	1.7 ± 1.24	<0.001
Inadequate bowel preparation, *n* (%)	29 (16.1)	12 (20.0)	7 (11.7)	10 (16.7)	0.458
Inadequate right‐colon preparation, *n* (%)	1 (0.5)	1 (1.7)	0 (0.0)	0 (0.0)	0.366

The mean number of bowel movements after PEG‐Asc ingestion was significantly lower in Group C (PLRD + 1.0 L PEG‐Asc) compared with Groups A and B (*p* < 0.001). Similarly, the mean intraluminal bubble score (0–6) was significantly lower in Group C, indicating less foaming during insertion (*p* < 0.001). Inadequate bowel preparation was defined as OBPS ≥5 for the entire colon. Inadequate right‐colon preparation was defined as a segmental OBPS score ≥3 in the right colon.

In contrast, several secondary cleansing indicators showed significant intergroup differences. The total number of bowel movements following PEG‐Asc ingestion differed markedly across the groups, with Group C exhibiting significantly fewer evacuations (8.9 ± 2.14) compared with Groups A (10.9 ± 2.20) and B (11.0 ± 2.38) (*p* < 0.001). Similarly, the amount of intraluminal bubbles—scored on a 0–6 scale—was lowest in Group C (1.7 ± 1.24), significantly reduced relative to Group A (2.3 ± 1.07) and Group B (1.4 ± 1.01) (*p* < 0.001). The number of cases with inadequate bowel preparation in each group was 12 (20.0%) in Group A, 7 (11.7%) in Group B, and 10 (16.7%) in Group C.

### 3.4 Factors Associated With Inadequate Cleansing

Risk factor analyses for inadequate bowel cleansing were performed for both the whole colon and specifically for the right colon. When inadequate cleansing of the whole colon was used as the outcome, none of the evaluated clinical or procedural variables demonstrated significant associations in the univariate analysis. Age, sex, BMI, prior colonoscopy, bowel movement frequency, the presence of diverticula, reduction in PEG‐Asc volume, and intake of the PLRD all showed non‐significant odds ratios, and no variables met criteria for multivariate modeling (Table 4).

**TABLE 4:** Risk factors for inadequate bowel cleansing in the whole colon.

	Univariate analysis			Multivariate analysis		
Variable	OR	Cl	*p*‐Value	OR	Cl	*p*‐Value
Age	1.018	0.981–1.056	0.338	—	—	—
Gender	1.292	0.543–3.075	0.647	—	—	—
BMI	1.025	0.936–1.122	0.600	—	—	—
Previous colonoscopy	1.186	0.490–2.872	0.827	—	—	—
BM less than once daily	0.835	0.310–2.247	0.793	—	—	—
Diverticulum	1.815	0.751–4.386	0.218	—	—	—
Reduction of PEG‐Asc volume	1.063	0.460–2.456	1.000	—	—	—
Intake of a prepackaged LRD	0.660	0.262–1.491	0.390	—	—	—

Univariate logistic regression analyses evaluating potential predictors of inadequate overall bowel preparation. No variables showed a statistically significant association with inadequate cleansing in the whole colon.

Abbreviations: BM, bowel movement; LRD, low‐residue diet.

In contrast, when inadequate cleansing of the right colon was examined, intake of the PLRD demonstrated a strong and statistically significant association. In the univariate analysis, consumption of the diet was associated with a markedly lower risk of inadequate cleansing (odds ratio [OR] 0.164, 95% confidence interval [CI] 0.079–0.341, *p* < 0.001). This association remained significant in the multivariate analysis, where intake of the prepackaged diet was independently protective against inadequate right‐sided cleansing (OR 0.119, 95% CI 0.043–0.327, *p* < 0.001). No other variables—including age, sex, BMI, diverticulosis, or reduction of PEG‐Asc volume—were significantly associated with right‐colon cleansing quality (Table 5).

**TABLE 5:** Risk factors for inadequate bowel cleansing in the right colon.

	Univariate analysis			Multivariate analysis		
Variable	OR	Cl	*p*‐Value	OR	Cl	*p*‐Value
Age	0.991	0.962–1.022	0.060	—	—	—
Gender	1.342	0.639–2.815	0.442	—	—	—
BMI	1.027	0.950–1.111	0.500	—	—	—
Previous colonoscopy	0.736	0.360–1.505	0.456	—	—	—
BM less than once daily	1.295	0.520–3.225	0.662	—	—	—
Diverticulum	0.875	0.379–2.018	0.837	—	—	—
Reduction of PEG‐Asc volume	0.486	0.222–1.065	0.071	—	—	—
Intake of a prepackaged LRD	0.164	0.079–0.341	<0.001	0.119	0.043–0.327	<0.001

Results of univariate and multivariate logistic regression analyses evaluating predictors of inadequate cleansing in the right colon segment. Among the tested variables, intake of a prepackaged low‐residue diet was independently associated with a significantly reduced risk of inadequate proximal cleansing.

These findings indicate that adherence to a standardized PLRD may meaningfully reduce the risk of inadequate preparation in the proximal colon. Given that inadequate cleansing of the right colon is particularly associated with missed advanced neoplasia, the potential benefit of this dietary approach may have important clinical implications.

## 4 Discussion

Various approaches have been explored to improve bowel preparation and patient acceptability [19, 20, 21, 22]. Nevertheless, the large volume of PEG‐Asc required in standard regimens continues to pose a substantial challenge for patient compliance in everyday practice. In this randomized exploratory study, we investigated whether a PLRD (Clear‐Through) could allow a further reduction in the volume of PEG‐Asc while preserving adequate bowel cleansing quality in a Japanese population. The results indicate that combining a PLRD with a reduced PEG‐Asc volume of 1.0 L appeared to provide bowel cleansing quality comparable to that of conventional regimens and is associated with favorable adherence, tolerability, and patient satisfaction.

Several earlier trials, such as the work from Taiwan by Chou and colleagues, demonstrated that structured dietary restriction can enhance bowel preparation and support modest reductions in purgative volume [16, 23, 24]. Rather than replicating prior studies, our trial extends existing evidence by evaluating this approach in a Japanese cohort with distinct dietary habits and body composition. Second, while earlier studies showed that a reduction to 1.5 L of PEG‐Asc was feasible, our findings suggest that the bowel preparation regimen used in this study, which includes a PLRD and adjunctive agents, may provide acceptable cleansing quality even with a reduced PEG‐Asc volume of 1.0 L. However, comparisons with previous studies based solely on preparation volume should be interpreted with caution, as our regimen differs in several important aspects, including the use of pre‐day picosulfate and a PLRD. In addition, bowel cleansing efficacy may vary depending on the type of cleansing agent used, reflecting differences in osmotic properties, volume requirements, and adjunctive components.

Although we evaluated a specific PLRD (Clear‐Through), differences in composition among PLRD products may influence bowel preparation outcomes. In particular, the PLRD used in this study contains maltooligosaccharides, which may facilitate bowel movements. Therefore, our findings should be interpreted with caution when applied to other PLRD formulations.

Beyond traditional cleansing endpoints, our study incorporated additional outcome measures—such as an intraluminal bubble score and detailed patient‐reported satisfaction metrics—that were not evaluated in prior trials. These supplementary assessments enabled a more comprehensive appraisal of preparation quality and patient experience. The PLRD groups, particularly the 1.0 L group, demonstrated favorable results in these domains, including reduced bubble burden and lower stool frequency, suggesting that dietary control may positively influence proximal colon cleanliness.

Adherence to the assigned regimen was high, and participants who received the PLRD reported greater satisfaction compared with their previous colonoscopy experiences. The simplified dietary instructions afforded by a prepackaged meal set may help patients comply more easily with preparation requirements, thereby reducing the psychological and physical burden associated with bowel cleansing [25, 26].

Importantly, intake of the PLRD emerged as an independent protective factor against inadequate cleansing of the right colon. Given the well‐established link between suboptimal proximal cleansing and missed advanced neoplasia, this finding underscores the clinical relevance of dietary standardization as an adjunct to bowel preparation [27, 28, 29, 30].

This study has several limitations. Patients with severe constipation, high BMI, or advanced age were excluded, which may limit generalizability. Satisfaction assessment relied on a non‐validated questionnaire and subjective recall. Moreover, as a single‐center exploratory trial, the sample size was limited. We did not perform a formal sample size calculation, and the number of 60 patients per group was determined pragmatically based on previous reports and the expected number of eligible patients at our institution. Therefore, the present trial should be regarded as a pilot exploratory randomized study rather than a fully powered confirmatory trial. Future multicenter studies with larger and more diverse populations are needed to validate these findings and to explore whether further optimization of dietary composition or purgative volume could improve preparation quality.

In conclusion, the incorporation of a PLRD into the bowel preparation regimen used in this study allowed a reduction in PEG‐Asc volume while maintaining comparable bowel cleansing quality in a Japanese population. Standardized dietary preparation may serve as a practical strategy to reduce patient burden and support high‐quality colonoscopy. Because this study was exploratory in nature, these findings should be interpreted cautiously and confirmed in larger studies specifically designed to evaluate non‐inferiority. Additional research will help clarify the role of dietary control within modern bowel preparation protocols.

## Author Contributions

Toshifumi Iida wrote the article and performed the statistical analyses. Toshifumi Iida, Yoshiaki Kimoto, and Ken Ohata conceived and designed the study. Etsuko Yamabe, Miuzen Kanamori, Susumu Banjoya, Tomoya Kimura, Koichi Furuta, Shinya Nagae, Hiroshi Yamazaki, Nao Takeuchi, Shunya Takayanagi, Yuki Kano, Kohei Ono, Ryoju Negishi, Yohei Minato, and Hideyuki Chiba provided patient management. Ken Ohata provided the final approval of the article.

## Conflicts of Interest

The authors declare no conflicts of interest.

## Funding

The authors have nothing to report.

## Ethics Statement

All procedures followed have been performed in accordance with the ethical standards laid down in the Declaration of Helsinki and its later amendments. The study was conducted with the approval of the Institutional Review Board of NTT Medical Center Tokyo (IRB No. 000200016279‐0).

## Consent

Informed consent was obtained from all participants.
